# Simultaneous Mini-ECIRS and low-energy TFL endopyelotomy for recurrent UPJO with pelvic renal calculus: A case report

**DOI:** 10.1016/j.eucr.2025.103190

**Published:** 2025-08-31

**Authors:** Manapol Rujithamkul, Kun Sirisopana, Surawach Piyawannarat, Yada Phengsalae, Premsant Sangkum, Wisoot Kongchareonsombat, Chinnakhet Ketsuwan

**Affiliations:** Division of Urology, Department of Surgery, Faculty of Medicine Ramathibodi Hospital, Mahidol University, Bangkok, Thailand

**Keywords:** Mini-ECIRS, Thulium fiber laser, Ureteropelvic junction obstruction

## Abstract

Recurrent ureteropelvic junction obstruction (UPJO) with renal calculi poses a surgical challenge. Miniature Endoscopic Combined Intrarenal Surgery (mini-ECIRS) with low-wattage Thulium Fiber Laser (TFL) enables simultaneous stone clearance and precise endopyelotomy. We report a case of a 51-year-old woman with recurrent UPJO and renal stones treated using mini-ECIRS and TFL-assisted retrograde endopyelotomy. Dual endoscopic access allowed effective stone fragmentation and accurate UPJ incision. Operative time was 75 min, with no complications. This case supports mini-ECIRS with low-energy TFL as a safe, single-session treatment for recurrent UPJO with renal stones.

## 1 Introduction

Ureteropelvic junction obstruction (UPJO) is a well-documented condition characterized by impaired urine drainage from the renal pelvis into the ureter. If undiagnosed or inadequately managed, UPJO can lead to progressive hydronephrosis and irreversible renal function impairment. The coexistence of renal calculi with UPJO is relatively common, with prevalence rates ranging from 16 % to 30 %.^1^^,^^2^

With advancements in endoscopic instruments and surgical techniques, combined endoscopic approaches have gained widespread acceptance worldwide. These minimally invasive methods have demonstrated clear advantages, particularly in complex clinical scenarios.^3^, ^4^, ^5^, ^6^ The principle of enhanced visualization using dual scopes and a dual irrigation system allows for more precise incisions with improved clarity during endopyelotomy, significantly contributing to better surgical outcomes. In this report, we describe our initial experience using mini-endoscopic combined intrarenal surgery with low-wattage thulium fiber laser technology to successfully manage renal stones and recurrent UPJO in a patient with a history of failed pyeloplasty.

## 2 Case report

**Fig. 1:**
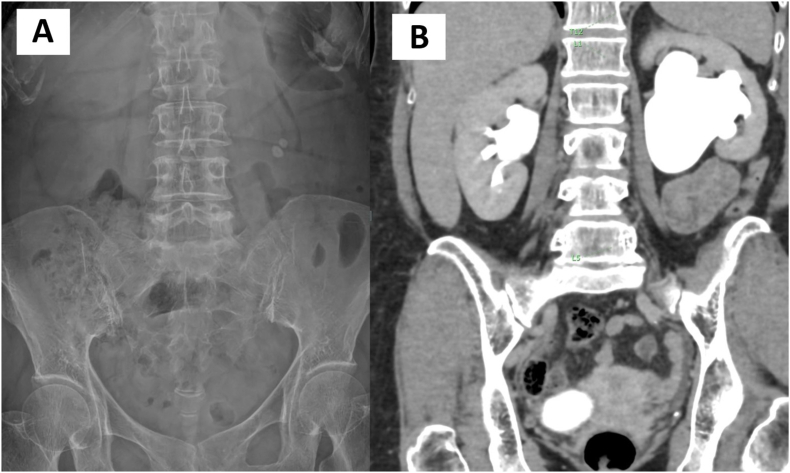
A – Abdominal X-ray showing two left renal pelvic stones; B – Computed tomography scan demonstrating severe left hydronephrosis with recurrent ureteropelvic junction obstruction.

A 51-year-old woman with a history of left ureteropelvic junction obstruction (UPJO) previously treated with laparoscopic dismembered pyeloplasty a decade earlier presented to our clinic with recurrent urinary tract infections and progressive hydronephrosis. Urinalysis revealed microscopic hematuria. Initial evaluation revealed a serum creatinine level of 0.55 mg/dL and two left renal calculi detected by abdominal X-ray (Fig. 1A). Contrast-enhanced computed tomography imaging showed severe left hydronephrosis with recurrent UPJO (Fig. 1B) and two non-obstructing renal pelvic stones measuring 1.0 cm and 0.7 cm, with densities of approximately 1100 Hounsfield units. A diuretic renogram (99mTc-MAG3) demonstrated delayed drainage with a T½ of 25 minutes. After multidisciplinary consultation and a patient-centered discussion on therapeutic options, mini-endoscopic combined intrarenal surgery (mini-ECIRS) combined with very low-energy thulium fiber laser (TFL) endopyelotomy was selected as the optimal treatment.

Prophylactic intravenous cefuroxime was administered under general anesthesia, and the patient was positioned in the Galdakao-modified supine Valdivia position. Rigid cystourethroscopy was initially performed, and a hybrid guidewire was advanced into the pelvicalyceal system. A disposable digital flexible ureteroscope (8.4 Fr) was introduced using the Seldinger technique without complications. However, endoscopic visualization of the renal pelvis stones was impeded by recurrent UPJO.

Percutaneous renal access was achieved via ultrasound-guided puncture using an 18-gauge needle. Following tract dilation, a ClearPetra nephrostomy sheath was positioned, establishing a 16 Fr working channel. A 12 Fr nephroscope was introduced, and laser lithotripsy was performed using a 60 W TFL system (SOLTIVE™, Olympus) with a 550-μm fiber at 1.5 J and 20 Hz settings. Effective stone fragmentation and removal were facilitated by integrated suction.

**Fig. 2:**
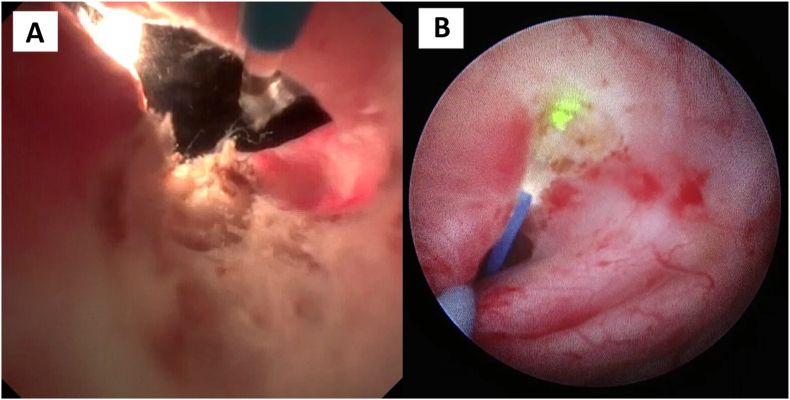
A – Retrograde view via flexible ureteroscopy; B – Antegrade view using a rigid nephroscope.

Subsequently, retrograde flexible ureteroscopy facilitated precise posterior incision of the UPJO from the urothelium to periureteral fat (Fig. 2A), guided by simultaneous antegrade visualization using a rigid nephoscope (Fig. 2B). The incision was conducted using low-energy TFL settings (1 J, 2 Hz).

**Fig. 3:**
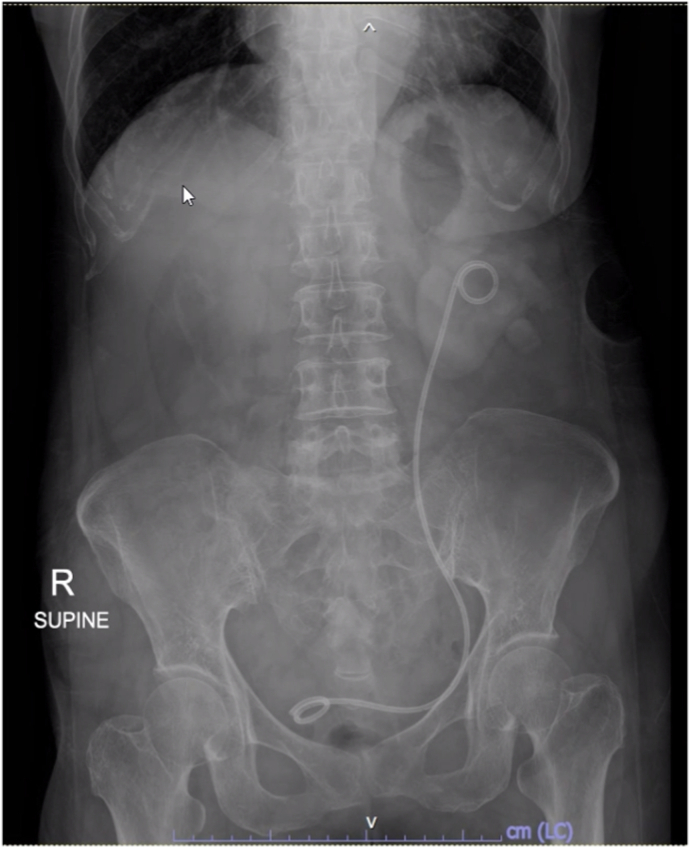
Postoperative abdominal radiograph showing complete stone clearance.

The total operative time was 75 min, including 20 min for stone fragmentation and 15 min for endopyelotomy. Postoperative imaging confirmed complete stone clearance and appropriate placement of an 8 Fr DJ stent and a 12 Fr nephrostomy tube (Fig. 3). The postoperative recovery was uneventful, and the patient was discharged without complications. The nephrostomy tube was removed one day postoperatively, and the DJ stent was removed cystoscopically at 6 weeks. Stone analysis was not performed, as the patient declined further testing due to financial considerations.

## 3 Discussion

Managing recurrent UPJO complicated by renal stones is particularly challenging, especially in patients with prior surgical interventions, such as laparoscopic or open pyeloplasty. Dense scar tissue complicates surgical reintervention, increasing the risk of bleeding, injury to surrounding structures, and prolonged recovery periods. Our case exemplifies the successful application of mini-ECIRS combined with TFL in overcoming these challenges.^7^

Historically, open pyelolithotomy combined with pyeloplasty represented the standard of care but carried significant morbidity, including postoperative pain, prolonged hospitalization, and potential incisional hernias.^8^ The development of miniaturized endoscopic techniques, such as mini-ECIRS, presents a minimally invasive alternative. The use of smaller-caliber instruments minimizes renal parenchymal trauma and may therefore reduce the risk of major hemorrhage, a complication more commonly encountered with conventional standard-caliber nephroscope techniques.^9^, ^10^, ^11^ This approach allows simultaneous antegrade and retrograde access, enhancing visualization and facilitating precise maneuvers within the collecting system. Consequently, both stone removal and precise incision of the UPJO can be efficiently achieved in a single session.^12^, ^13^, ^14^ Moreover, in the present case, a mini-nephroscope was employed for the antegrade approach rather than the conventional standard nephroscope.

The integration of TFL technology contributed to procedural success by enabling precise tissue cutting and efficient stone fragmentation, minimizing collateral thermal injury. Its controlled energy delivery is particularly beneficial in endopyelotomy procedures, in which accurate incision is critical. In this case, very low-energy TFL settings (1 J, 2 Hz) enabled precise incisions under dual visualization, enhancing safety and accuracy. Additionally, the TFL's fine fragmentation and dusting capabilities, combined with active suction through the sheath, ensured complete stone clearance^15^, ^16^, ^17^, ^18^—a critical step prior to performing the endopyelotomy incision. This process minimizes the risk of residual stone fragments that could lead to recurrent scarring at the UPJO site.

While our outcome was favorable, the long-term patency rates of endopyelotomy procedures for recurrent UPJO vary. Nonetheless, the precision and visualization afforded by TFL and mini-ECIRS may positively influence long-term success. Further studies with larger patient cohorts are necessary to validate long-term benefits and to define broader indications for this promising combined approach.

## 4 Conclusion

This case highlights the efficacy of mini-ECIRS combined with TFL-assisted endopyelotomy for managing recurrent UPJO and concurrent renal calculi. The integration of these advanced minimally invasive techniques offers precise, effective treatment, promising improved outcomes, and reduced patient morbidity. Further research is warranted to confirm long-term efficacy and recurrence rates.

## CRediT authorship contribution statement

**Manapol Rujithamkul:** Writing – original draft, Project administration, Formal analysis, Data curation, Conceptualization. **Kun Sirisopana:** Methodology, Investigation. **Surawach Piyawannarat:** Methodology, Investigation. **Yada Phengsalae:** Methodology, Investigation. **Premsant Sangkum:** Validation, Supervision. **Wisoot Kongchareonsombat:** Validation, Supervision. **Chinnakhet Ketsuwan:** Writing – review & editing.

## Ethical approval

This case report was conducted in accordance with the ethical standards of the institution and the 1964 Helsinki Declaration and its later amendments or comparable ethical standards. The ethical approval number is COA No. MURA2025/448. Informed consent was obtained from the patient included in this case report.

## Funding

This research received no specific grant from any funding agency in the public, commercial, or not-for-profit sectors.

## Declaration of competing interests

The authors declare that they have no conflicts of interest.
